# Metronidazole-Induced Acute Cerebellitis in a Young Patient: Unusual Onset, Delayed Remission, and Characteristic Imaging Features

**DOI:** 10.7759/cureus.56098

**Published:** 2024-03-13

**Authors:** Venkat Reddy, Sunil Kumar, Sourya Acharya, Jasleen Kakkad, Mamtha Jadhav

**Affiliations:** 1 Department of General Medicine, Jawaharlal Nehru Medical College, Datta Meghe Institute of Higher Education and Research, Wardha, IND; 2 Department of Otolaryngology - Head and Neck Surgery/General Medicine, Jawaharlal Nehru Medical College, Datta Meghe Institute of Higher Education and Research, Wardha, IND

**Keywords:** postural instability, dysarthria, weakness, drug-induced acute cerebellitis, metronidazole

## Abstract

Metronidazole-induced acute cerebellitis is an exceptionally rare condition resulting from severe adverse reactions to metronidazole, a medication generally employed in the management of infections caused by anaerobic microbes. Although neuropathy has been linked to metronidazole use, reports of acute cerebellitis are infrequent. The neurological effects associated with metronidazole can include weakness, dysarthria, postural instability, seizures, giddiness, vertigo, ataxia, confusion, encephalopathy, headaches, and tremors. The onset of cerebellitis can vary, occurring as early as one day or after several weeks of metronidazole treatment. This article presents a case of a young girl who presented to us with weakness in both upper and lower limbs, dysarthria, and postural instability after exposure to 12 grams of metronidazole (suicidal, 30 tablets of 400 mg). With the above-mentioned complaints, the patient was advised of magnetic resonance imaging of the brain, which showed the features of cerebellitis.

## Introduction

Metronidazole-induced acute cerebellitis is a rare phenomenon linked to the antibiotic metronidazole [1]. Metronidazole is a synthetic 5-nitroimidazole antibiotic that is widely used as a medication to fight protozoa and anaerobic bacteria. Since it has been used for more than 50 years to treat infections, metronidazole is still the medication of choice for treating anaerobic bacterial infections with a low risk of resistance. It is also used to prevent or delay clinical recurrence in Crohn's disease. Metronidazole is known for its effective cellular penetration, including access to the central nervous system and cerebrospinal fluid [2].

At appropriate doses, it is generally considered harmless. However, if it is used in excessive amounts or for an extended period, it can lead to peripheral neuropathies and cerebellar dysfunction. The precise incidence of metronidazole-induced acute cerebellitis remains unknown, but prior research has examined the brain changes associated with metronidazole neurotoxicity [3].

## Case presentation

A young woman visited the outpatient clinic complaining of weakness in both the upper and lower limbs, ataxia, dysarthria, and postural instability. She gave the history of consumption of metronidazole tablets around 12 grams (30 tablets of 400 mg). There was no history of suicidal tendencies in the past. She was not taking any other medications such as antiepileptic drugs. There was no family history of hypertension, diabetes, or alcohol abuse.

During the examination, the patient had a normal body temperature, with a blood pressure of 110/70 mmHg, a heart rate of 78 bpm, a respiratory rate of 18 breaths per minute, and oxygen saturation (SpO2) at 100%. The patient had no pallor, no jaundice, and no cyanosis, clubbing, or lymphadenopathy. Her BMI was 22.9 kg/m2. Her speech was scanning type. The patient had minor dysmetria in both arms, as shown by coordination tests, including a finger-to-nose exam. During the gait evaluation, the patient was falling in all directions. She was also having truncal ataxia and horizontal nystagmus in both eyes. The tone was normal in all four limbs. Her superficial and deep tendon reflexes were normal. Power was grade 5/5 in all four limbs and other neurological investigations revealed no abnormality.

Her laboratory investigations revealed hemoglobin of 12.2 gm/dL, mean corpuscular volume (MCV) of 90.8 fL (femtoliter), and white blood cell (WBC) count of 6000/cumm, as shown in Table 1. Her kidney function test, liver function test, serum protein, lipid profile, random blood sugar, and other investigations were within normal limits. The lumbar puncture showed 4 milliliters of transparent and uncolored cerebrospinal fluid, in which the level of protein was 52 mg/dL (n = 15-45 mg/dL) and WBC count was 10 cells/cumm (n = 0-5 cells/cumm), both of which were slightly increased. Cerebrospinal fluid (CSF) sugar was 60 mg/dL (n = 50-80 mg/dl), differential leukocyte count (DLC) was 90% lymphocytes and 10% polymorphs, and the wet film showed 1-2 lymphocytes, 0-1 polymorphs, and occasional RBCs/high power field. No acid-fast bacilli were seen in the Ziehl-Neelsen (ZN) stain and gram statin did not show growth of any organism. Parallel blood sugar was 92 mg/dL.

**Table 1:** Investigation profile of the patient on the day of admission. Hb: hemoglobin; TLC: total leucocyte count; MCV: mean corpuscular volume; ALT: alanine aminotransferase; AST: aspartate aminotransferase; RBS: random blood sugar; TSH: thyroid stimulating hormone.

Lab parameters	Observed value	Normal range
Hb	12.2 gm%	13-17 gm%
MCV	90.8 fL	83-101 fL
TLC	6000 cells/cu mm	4000-10000 cells/cu mm
Platelets	3.2 lakhs/ cu mm	1.5-4.1 lakhs/ cu mm
Urea	38 mg/dL	19-43 mg/dL
Creatinine	1.0 mg/dL	0.66-1.25 mg/dL
Sodium	132 mmol/L	137-145 mmol/L
Potassium	4.1 mmol/L	3.5-5.1 mmol/L
Calcium	9.2 mg/dL	8.4-10.2 mg/dl
Magnesium	2.0 mg/dL	1.6-2.3 mg/dL
Phosphorous	2.9 mg/dL	2.5-4.5 mg/dL
Uric acid	4.2 mg/dL	3.5-8.5 mg/dL
Alkaline phosphatase	130 U/L	38-126 U/L
ALT	22 U/L	<50 U/L
AST	48 U/L	17-59 U/L
Albumin	4.4 g/dL	3.5-5 g/dL
Total bilirubin	0.9 mg/dl	0.2-1.3 mg/dl
Conjugated bilirubin	0.3 mg/dl	0.0-0.3 mg/dl
Unconjugated bilirubin	0.6 mg/dl	0.0-1.1 mg/dl
RBS	134 g/dL	90-140 g/dL
Vitamin B12	800 pg/ml	239-931 pg/ml
Vitamin D	8.27 ng/ml	<20 ng/ml
TSH	0.522 mIU/ml	0.465-4.68 mIU/ml

**Figure 1:**
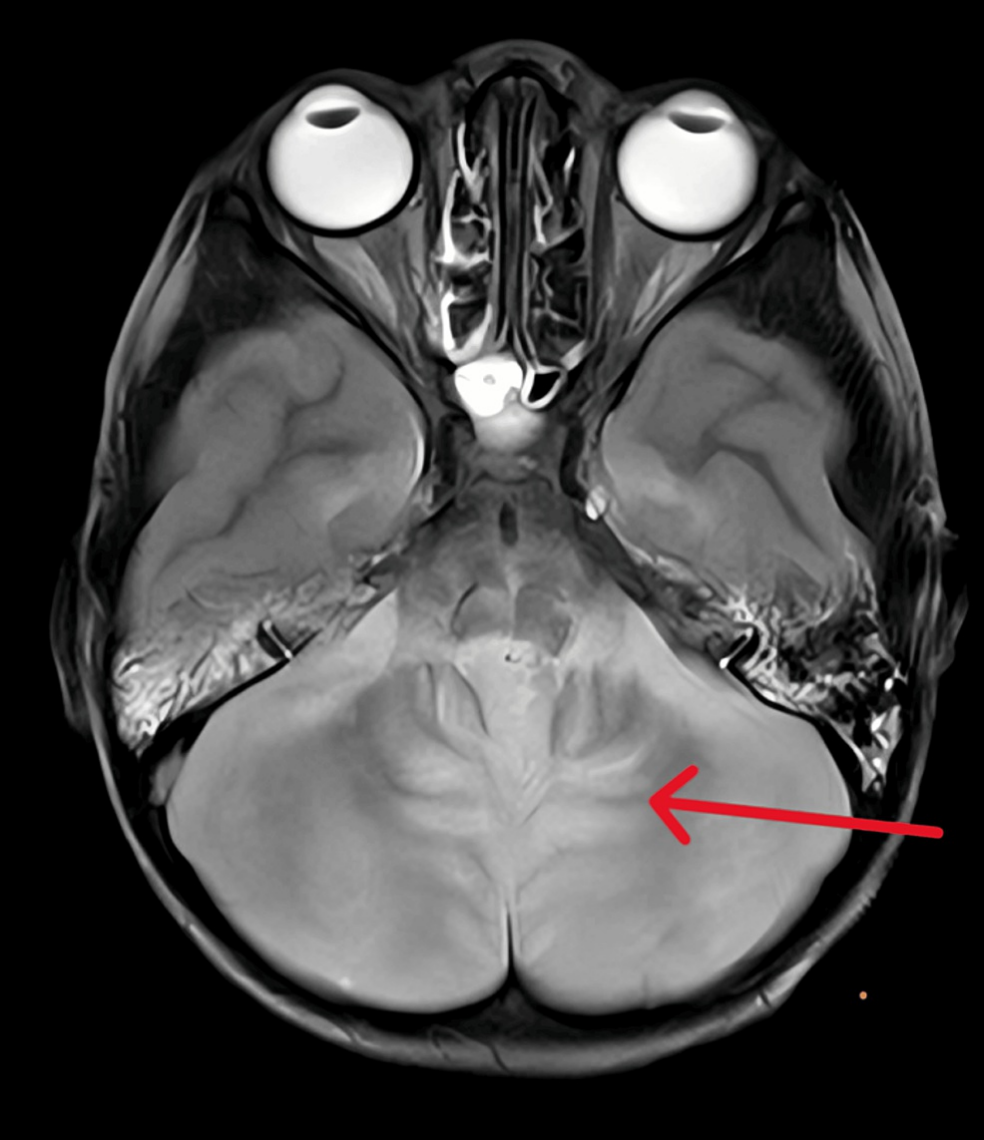
MRI (T2-weighted image/FLAIR) axial view showing mild edema with diffuse hyperintensity in the cerebellum (shown with red arrow) with associated leptomeningeal enhancement suggestive of acute cerebellitis. FLAIR: fluid-attenuated inversion recovery.

In view of neurological manifestations, magnetic resonance imaging (MRI) was planned, which has been described in Figure 1.

With such symptoms, immediate gastric lavage was given with normal saline. As metronidazole overdose does not have an antidote, forced alkaline diuresis was done by giving sodium bicarbonate in the dose of 1 mEq/kg in 0.9% normal saline, 100 ml intravenously in initial 30-35 minutes, followed by sodium bicarbonate 75 mEq and 25 mEq of potassium in 5% dextrose, and 500 ml in next eight hours. Injection of furosemide 20 mg thrice a day was given for five days, and then tapered and continued orally for the next three days. Electrolyte balance was confirmed before initiating diuresis. Injectable mannitol was given to reduce cerebral edema. As the patient appeared to be confused, we administered a high dose of IV methylprednisolone pulse therapy (500 mg/day).

Her dysarthria considerably improved on the fourth day of her hospital stay. On the second day after starting glucocorticoid therapy, she was allowed to walk, albeit with some balance problems. The glucocorticoid was gradually tapered starting on the fifth day of admission at a dose of 40 mg once a day for five days, followed by 20 mg once a day for five days, 10 mg once a day for five days, and then stopped. The patient was asymptomatic after 15 days.

## Discussion

One of the cornerstone medications for the management of microaerophilic, protozoal, and anaerobic bacterial infections is metronidazole. Microorganisms that are facultative anaerobic are cytotoxic to it [4]. When metronidazole interacts with DNA, it produces strand breaks and a loss of helical deoxyribonucleic acid (DNA) structure, resulting in cell death in vulnerable animals. Metronidazole also diffuses into the body. It is used to treat a variety of conditions, including intra-abdominal infections, lower respiratory tract infections, skin structure infections, meningitis, amebiasis, bacterial septicemia, bone and joint infections, brain abscess, endocarditis, endometritis, and bacterial vaginosis [4].

The Food and Drug Administration (FDA) has approved metronidazole for the treatment of anaerobic bacterial infections caused by *Bacteroides* species, *Fusobacterium* species, *Clostridium* species, *Gardnerella vaginalis*, *Helicobacter pylori*, *Prevotella* species, *Porphyromonas* species, and *Bilophila wadsworthia*, as well as protozoal infections such as *Trichomonas vaginalis*, *Entamoeba histolytica*, *Giardia lamblia*, blastocysts, and *Balantidium coli*. For rosacea, topical metronidazole is recommended. For bacterial vaginosis, it is administered intravaginally [4,5].

Confusion, peripheral neuropathy, metallic taste, nausea, vomiting, and diarrhea are among the main side effects of metronidazole [6]. Metronidazole, generally considered safe, has few adverse effects, with a range of neurological complications associated with its use that are often overlooked. These complications encompass various syndromes such as cerebellar dysfunction, encephalopathy, seizures, and issues with optic, autonomic, and peripheral nervous systems. Due to the reversible nature of many of these neurological effects upon cessation of the drug, instances of metronidazole-induced neurotoxicity may be underreported or unrecognized [7].

Cerebellar dysfunction, particularly ataxia (other causes of ataxia) and dysarthria, is the most prevalent presentation among patients affected with metronidazole-induced cerebellitis [8-10]. With prolonged self-medication drug use in this particular patient, the problem emerged shortly after initiating metronidazole treatment. As per previous literature reviews, the time it takes for symptoms to remit after discontinuing metronidazole can vary, spanning from a few days to several weeks. The median duration for the restoration of central nervous system function is reported to be 23.3 days [11].

In our specific case, MRI findings using T2-fluid-attenuated inversion recovery (FLAIR) images revealed mild edema with diffuse hyperintensity in the cerebellum, with associated leptomeningeal enhancement suggestive of acute cerebellitis. The most likely mechanism of metronidazole's neurotoxicity is that its ingredients produce hydrogen peroxide and superoxide radicals, which in turn cause intramyelinic edema and myelin vacuoles [12].

Typically, metronidazole-induced brain damage is reversible, with symptoms resolving within a few days of discontinuing the drug. Metronidazole, a widely used antibiotic, is associated with various neurological adverse effects, albeit rare. Cerebellitis following metronidazole use is an infrequent yet significant complication. However, in our case, the patient's condition improved four days after discontinuation. Notably, Li et al. introduced a novel approach by using methylprednisolone, marking the first instance of such treatment to avert potentially fatal complications. Methylprednisolone serves to alleviate inflammation and edema in tissue, hence enhancing perfusion in microcirculation and local cerebral blood flow. Additionally, its antioxidant properties play a crucial role in protecting neurons, stabilizing lysosomes, suppressing free radicals, reducing axonal edema, and increasing GABA (gamma-aminobutyric acid) release [13,14]. It is worth mentioning that there are no prior case reports of steroids being employed in such cases. This underscores the importance of early recognition of metronidazole-induced cerebellitis, prompt discontinuation of the offending drug, and considering alternative treatment options to prevent fatal consequences.

Physicians ought to have a high degree of mistrust regarding this rare adverse effect, especially in cases where other etiologies have been ruled out. To fully understand the exact causes and best practices for managing metronidazole-induced cerebellitis, more study is necessary [15].

One limitation of this report is that there was no long-term follow-up done. Longitudinal studies with larger sample sizes are required to better perceive the incidence, risk factors, and outcomes related to metronidazole-induced cerebellitis.

## Conclusions

This report presented metronidazole-induced acute cerebellitis, which recovered completely after proper management. It is conceivable that the progression of metronidazole-induced acute cerebellitis may be linked to delayed toxicity or sustained effects in the bloodstream or the brain. Further research is warranted to explore the potential neuroprotective mechanisms of glucocorticoids (possibly referring to methylprednisolone) in the context of metronidazole-induced acute cerebellitis.
